# An immunohistochemistry-based classification of colorectal cancer resembling the consensus molecular subtypes using convolutional neural networks

**DOI:** 10.1038/s41598-025-03618-z

**Published:** 2025-05-31

**Authors:** Tuomas Kaprio, Jaana Hagström, Jussi Kasurinen, Ioannis Gkekas, Sofia Edin, Ines Beilmann-Lehtonen, Karin Strigård, Richard Palmqvist, Ulf Gunnarson, Camilla Böckelman, Caj Haglund

**Affiliations:** 1 Department of Surgery, University of Helsinki and Helsinki University Hospital, Haartmaninkatu, 00290 Helsinki, Finland; 2 Research Programs Unit, Translational Cancer Medicine, University of Helsinki, Helsinki, Finland; 3 Department of Pathology, University of Helsinki and Helsinki University Hospital, Helsinki, Finland; 4 Department of Surgical and Perioperative Sciences, Umeå University, Umeå, Sweden; 5 Department of Medical Biosciences, Pathology, Umeå University, Umeå, Sweden; 6 Department of Oral Pathology and Radiology, University of Turku, Turku, Finland; 7 Department of Diagnostics and Intervention, Umeå University, Umeå, Sweden

**Keywords:** Colorectal cancer, Immunohistochemistry, Consensus molecular subtypes, Prognosis, Convoluted neural network, Colorectal cancer, Tumour biomarkers

## Abstract

Colorectal cancer (CRC) represents a major global disease burden with nearly 1 million cancer-related deaths annually. TNM staging has served as the foundation for predicting patient prognosis, despite variation across staging groups. The consensus molecular subtype (CMS) is a transcriptome-based system classifying CRC tumors into four subtypes with different characteristics: CMS1 (immune), CMS2 (canonical), CMS3 (metabolic), and CMS4 (mesenchymal). Transcriptomics is too complex and expensive for clinical implementation; therefore, an immunohistochemical method is needed. The prognostic impact of the immunohistochemistry-based four CMS-like subtypes remains unclear. Due to the complexity and costs associated with transcriptomics, we developed an immunohistochemistry (IHC)-based method supported by convolutional neural networks (CNNs) to define subgroups that resemble CMS biological characteristics. Building on previous IHC-classifiers and incorporating β-catenin to refine differentiation between CMS2- and CMS3-like profiles, we categorized CRC tumors in a cohort of 538 patients. Classification was successful in 89.4% and 15.9% of tumors were classified as CMS1-like, 35.1% as CMS2-like, 38.7% as CMS3-like, and 11.7% as CMS4-like. CMS2-like patients exhibited the best overall survival (*p* = 0.018), including when local and metastasized disease were analyzed separately. Our method offers an accessible and clinically feasible CMS-inspired classification, although it does not serve as a replacement for transcriptomic CMS classification.

## Introduction

Colorectal cancer (CRC) represents a major disease burden globally with a rising incidence expected to reach 2.2 million by 2030 and nearly one million cancer-related deaths occurring annually^1,2^. Staging using the tumor-node-metastasis (TNM) system has remained the basis for treatment decisions alongside molecular markers: microsatellite instability (MSI), the *BRAF* mutation, and more recently *KRAS/NRAS* mutation status. Even when using these classification tools, CRC has differing outcomes within each subgroup. We thus need better methods to identify important CRC prognostic subgroups, providing possibilities for treating patients according to the specific biological properties of their specific tumor.

Almost ten years ago, the Colorectal Cancer Subtyping Consortium^3^ proposed, based on six previous gene expression–based classification systems^4–9^, a consensus-based molecular subtype (CMS) classification for CRC. CRC was divided into four molecular subtypes based on their molecular and genetic profiles: CMS1 (microsatellite instable [MSI] immune), CMS2 (canonical), CMS3 (metabolic), and CMS4 (mesenchymal)^3^. A recent meta-analysis of the clinical value of CMSes found that CMS4 accompanied the worst overall survival (OS) among patients with local disease (stages I–III) possibly due to the mesenchymal and invasive traits of these tumors, resulting in metastatic dissemination^10^. In metastatic disease, CMS1 had the worst OS driven by its association with the *BRAF* mutation^10,11^. CMS2 had the most favorable prognosis in metastatic disease^10^.

A major clinical problem is that the gene expression–based analysis is, due to its inherent complexity and cost, unfeasible for routine clinical use or for the analysis of large patient series in research settings. Several CMS classification tools applicable to formalin-fixed, paraffin-embedded CRC tissue samples based on either gene expression or immunohistochemistry (IHC) have, therefore, been proposed. The CMScaller is a classification system based on the identification of enriched gene expression markers in each subtype^12^. Because genetic alterations cause changes to tumors, detectable as changes in protein expressions, IHC may prove applicable to clinical use. Phenotypic subtyping based on the infiltration of immune cells, stromal invasion, and proliferation subtype was presented by Roseweir et al.^13^. We previously identified an association between CMS using this classification, clinicopathological variables, and survival, with the immune subtype associating with the best prognosis^14^. Another IHC panel for CMS-like classification was presented by Trinh et al.^15^, where MSI-high tumors were classified as CMS1-like and the remaining tumors based on the immunoexpression of the five proteins (CDX2, HTR2B, ZEB1, Cytokeratin(KER), and FRMD6) fell into subgroups CMS2/3-like and CMS4-like. The lack of a distinction between CMS2-like and CMS3-like may be solvable using β-catenin IHC^16^.

A recent meta-analysis^10^ estimated the impact of different CMS classification methods for defining the CMS subtypes and evaluated whether the differences in the techniques used will lead to different prognostic and predictive results. The majority of cohorts in that meta-analysis were classified based on gene expression data. Using IHC, four cohorts were classified into three CMS-resembling subtypes (CMS1, CMS2/3, and CMS4). The inability to differentiate between CMS2 and CMS3- caused the most pronounced differences when comparing IHC-based methods to gene expression–based methods. Specifically, the outcome of both the predictive and prognostic evaluations of the CMS classifications were similar for IHC- and gene expression–based data^10^. Therefore, it was concluded that CMS classification methods are robust and do not depend upon the specific method used, a major advantage for its future clinical implementation^10^.

In this study, we applied the IHC-based method described by Trinh et al.^15^, supplementing it with the addition of staining for β-catenin as described by Li et al.^16^. We categorized CRC tumors into four CMS-resembling subtypes on the largest patient cohort reported thus far. Our study aims to establish a clinically feasible alternative. While our classification system aligns with the characteristics of CMS, it is not a direct replacement for transcriptomic-based classification. The four CMS-resembling subtypes were evaluated in terms of their associations with clinicopathological parameters and patient prognosis.

## Materials and methods

### Study population

This cohort consisted of 538 CRC patients surgically treated between 1998 and 2005 at the Department of Surgery, Helsinki University Hospital. Clinical data were obtained from patient records and survival data were provided by the Finnish Population Registration Center and Statistics Finland. The median age of patients at diagnosis was 69.0 (range 32.0–96.1), and the median length of overall survival (OS) was 6.5 years (range 0–19.5).

### Ethical approval

The handling of tissue samples and patient data was approved by the Surgical Ethics Committee of Helsinki University Hospital (Dnro HUS 226/E6/06, extension TMK02 §66 17.4.2013) and the Finnish Medicines Agency (Dnro FIMEA/2021/006901 28.12.2021). The study was conducted in accordance with the Declaration of Helsinki.

### Preparation of tumor tissue microarrays

Paraffin blocks of tumor samples from surgical specimens fixed in formalin were collected from the archives of the Department of Pathology at the University of Helsinki. An experienced pathologist re-evaluated hematoxylin- and eosin-stained sections to confirm the diagnosis and marked representative areas of the tumors. Four 1.0-mm-diameter punches were taken from each tumor block using a semiautomatic tissue microarray instrument (TMA) (Beecher Instruments, Silver Spring, MD, USA).

### Immunohistochemical protocol

Tissue blocks were freshly cut into 4-µm sections, fixed on slides, and dried at 37 °C for 12 to 24 h. Slides were treated in a PreTreatment module (Agilent Dako, CA, USA) with a pH 9 retrieval solution (Envision Flex target retrieval solution, DM828, Agilent Dako) for 15 min at 98 °C for antigen retrieval. We stained sections with Autostainer 480S (LabVision Corp. Fremont, CA, USA) using Dako REAL EnVision Detection System, Peroxidase/DAB + , Rabbit/Mouse. First, we treated slides with Envision Flex peroxidase-blocking reagent SM801 for 5 min to block endogenous peroxidases. The antibodies and dilutions used for IHC staining appear in Supplementary Table 1. Subsequently, all slides underwent a 30-min incubation period with a peroxidase-conjugated EnVision Flex/HRP (SM802) rabbit/mouse (ENV) reagent. Slides were visualized using DAB chromogen (EnVision Flex DAB, DM827) for 10 min. Mayers hematoxylin (S3309, Dako) was used for counterstaining.

### Determining the CMS-resembling subtypes

#### Defining CMS1-like subtype

The MMR status—proficient or deficient—was evaluated using IHC analyses of all four protein products of genes involved in the DNA MMR system (MLH1, MSH2, PMS2, and MSH6) as reported elsewhere^17^. Tumors with a dMMR status were classified as CMS1-like.

#### Categorizing the CMS2/3 and CMS4 resembling subgroups using the IHC-CMS classifier

Four individual TMA spots were scored using CDX2, FRMD6, HTR2B, ZEB1, and KER immunohistochemical markers, with convoluted neural networks (CNNs) assisting in quantitative analysis, as described in detail in the online classification tool (crcclassifier.shi-nyapps.io/appTesting/)^15^. To clarify, the online classifier does not employ CNN directly; instead, it serves as a reference for staining interpretation. The training and validation of CNNs are discussed in detail below. The CMS-resembling status was calculated individually for each TMA spot. In the case of differences in the results between TMA spots from a specific tumor, the most frequent CMS-resembling status was chosen. In cases of equal amount of both CMS-resembling classes, the sample was considered as inconclusive (n = 3) and dismissed from further analysis.

#### Distinguishing between subgroups resembling CMS2 and CMS 3

Β-catenin was assessed for the intensity and percentage of cells with a positive nuclear staining in TMA spots from tumors classified as resembling CMS2/ CMS3 as in Li et al.^16^. The intensity was scored as 0–3 (0, negative; 1, low; 2, moderate; and 3, high) while the percentage was scored as 0–4 (0, negative; 1, 1–10%; 2, 11–50%; 3, 51–90%; 4, ≥ 90% of cells). A positive β-catenin record required nuclear staining with a score of ≥ 2 either in intensity or percentage. β-catenin-positive tumors were categorized as CMS2-like. In the case of differences in results between TMA spots from a specific tumor, the most frequent β-catenin status was chosen for further analysis. In cases involving inconclusive results we excluded the sample from further analysis.

### A semi-quantitative classification system using convoluted neural networks

Because interpreting thousands of individual TMA spots is laborious and prone to subjective human experience, we decided to use convoluted neural networks (CNNs) to assist with the interpretation of stainings in the online classifier. Four individual TMA spots were analyzed for CDX2, FRMD6, HTR2B, ZEB1, and KER expressions, supplemented by the use of CNNs. All cases were reviewed by an experienced pathologist when the TMA series were constructed. Individual samples were scored for both the cytoplasmic intensity and the percentage of KER. Intensity was scored using exact values. The percentage of KER was scored as the percentage of positively stained cells compared to negative cells in the tissue section, based on a calculation of the relative amount of epithelium. The nuclear staining of CDX2 and ZEB1 and the cytoplasmic staining of FRMD6 and HTR2B were analyzed in the tumor epithelial cells. The intensity and percentage of positive epithelial cells were calculated for CDX2 and FRMD6. The intensity of positive intra-tumoral epithelial cells was scored for HTR2B. Epithelial tumor cells were scored as either present or absent ZEB1, with a 2% cut-off to account for any potential false-positives. Each individual TMA spot’s probability of being an epithelial or mesenchymal type was counted separately by inputting the result from the CNNs into the online classifier calculation model. In cases involving different CMS-resembling classifications between TMA spots, we chose the most common. In cases where the results were inconclusive, we excluded the sample from further analysis (n = 3).

Stained TMA slides were digitized using the Panoramic 250 Flash3 whole-slide scanner (3D Histech, Budabest, Hungary) using a 20 × objective. The high-resolution (200 nm/pixel) digital whole-slide images obtained were then uploaded to the Aiforia Cloud v4.6 (Aiforia Inc., Cambridge, MA, USA) for image processing (cloud.aiforia.com).

Each deep learning-based model was trained on annotations (TK) from a subset of TMA spots. Annotations were made using a drawing tool provided by the graphical interface. The subset constituted approximately 5% of the available TMA spots, which were chosen to ensure capture of the variability in tissue morphology and in relation to image and staining quality across each dataset.

The models consist of multiple nested layers, where each subsequent layer only analyses pixels passed from the previous layer. Individual layers were put together to create a model capable of simultaneously detecting tissue areas and intensities. Each layer was trained using a growing number of annotations and iterations, until the model performed satisfactorily.

CNNs were trained to recognize, quantify, and measure the intensity depending upon the features of interest as defined above. Examples of areas are “epithelium” versus “tissue” or “cytoplasm” versus “nuclei” (see Supplementary Table 2 for details). Models were taught to analyze the intensity of the immunoreactivity, and yield the exact values for the intensity, which were rounded up to the nearest integer (0–0.499 to 0, 0.5–1.499 to 1, etc.) to fit the classification tool. For the percentage of the areas, we used exact values. The workflow for developing the CNNs appears in Supplementary Fig. 1.

### Validation of convoluted neural networks

The models were validated on an independent test set using a subset of tissue areas different from those upon which the model was trained. In total, 30 validation regions in 30 different patients’ tumors per layer were drawn by TK. Within these regions, the areas of interest were annotated by three independent human validators (JH, HL, and HK). The F1 score (the harmonic mean of precision and sensitivity) for each model versus each human validator was gathered for all validation regions and averaged across validators. The models produce exact regression values representing the intensity. The rounded values of these were compared to values provided by the validators, resulting in a percentage of matching values (matching intensity %). To determine the overall performance of each model, these measured values were compared against three validators and between validators.

### Statistical analysis

The Fisher’s exact test was used to test for associations between different CMS-resembling subtypes and clinicopathological parameters. The survival analysis was calculated using the Kaplan–Meier method and compared using the log-rank test. Overall survival was calculated from the day of surgery to the date of death or until the end of follow-up, while disease-specific survival (DSS) was calculated from the day of surgery until the date of death due to CRC or the end of follow-up. Univariate and multivariate survival analyses were calculated using the Cox proportional hazard models using the enter method. Only variables significant in the univariate analysis were entered into the multivariable model. Testing the Cox model assumption of a constant hazard ratio (HR) over time involved plotting the Schoenfeld residuals across time and testing for a correlation, with no relevant nonproportionality of HRs identified. We explored the possibility of interaction terms, identifying none. For all analyses, we considered *p* ≤ 0.05 as statistically significant, and all tests were two-sided. All statistical analyses were performed using SPSS version 27.0 (IBM SPSS Statistics, version 27.0 for Mac; SPSS Inc., Chicago, IL, USA, an IBM Company). To validate the CNN precision and sensitivity, values were acquired using the Aiforia image analysis software. Precision was calculated as the model’s analytical result area found within the pathologist’s annotation area per total area of the model’s analysis result area in a single validation area. Sensitivity was calculated as the pathologist’s annotation area found by the model’s analysis per total area of pathologist’s annotation in a single validation area. The F1 score represented the harmonic mean of precision and sensitivity.

## Results

### Immunohistochemistry

**Fig. 1:**
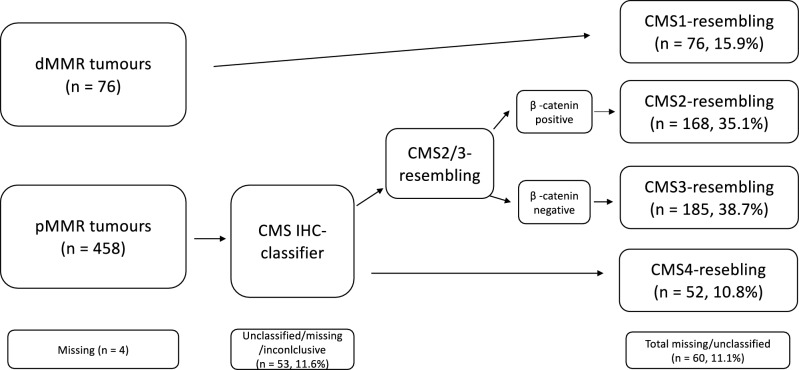
CMS-resembling classification based on IHC. First, the MMR status was used to identify patients belonging to the CMS1-resembling subtype. The CMS classifier then divided the remaining patients into the CMS2/3-resembling or CMS4-resembling subtypes. Finally, the CMS2/3-resembling group was divided based on the β-catenin staining.

Out of 538 patients, IHC-based CMS-resembling classification was successful for 481 patients (89.4%); 76 (15.9%) patient tumors were classified as CMS1-like, 168 as CMS2-like (35.1%), 185 as CMS3-like (38.7%), and 52 as CMS4-like (11.7%). Due to limitations in directly validating the CMS2-like vs CMS3-like division against a transcriptomic gold standard, our findings should be interpreted as defining an IHC-based prognostic classification system, rather than as a direct replication of the transcriptomic-based CMS. Figure 1 provides a flowchart of the study sample, while examples of positive IHC stainings appear in Supplementary Fig. 2.

### Performance of the convoluted neural networks

The F1 scores representing the precision and sensitivity exceeded 98% in a majority of the models, providing an excellent result. Matching of the intensity scores was better between model-to-validator than between validator-to-validator comparisons showing an acceptable performance (Supplementary Table 3).

### Association with clinicopathological variables

The associations between the CMS-resembling subtypes and the clinicopathological variables are summarized in Table 1. CMS1-like was associated with a non-mucinous histology (*p* = 0.001) and a right colon tumor location (*p* < 0.0001). CMS3-like occurred more often in elderly patients (*p* = 0.027), and CMS4-like was associated with a rectum tumor location (*p* < 0.0001).

**Table 1:** Association of clinicopathological characteristics and CMS-resembling subtypes.

Clinicopathological	CMS1-like	CMS2-like	CMS3-like	CMS4-like	*p* value*
Variable	76 (15.8%)	168 (34.9%)	185 (38.5%)	52 (10.8%)	
Age					
< 69	38 (50.0%)	93 (55.4%)	74 (40.0%)	28 (53.8%)	0.027
≥ 69	38 (50.0%)	75 (44.6%)	111 (60.0%)	24 (46.2%)	
Sex					
Female	41 (53.9%)	78 (46.4%)	83 (44.9%)	24 (46.2%)	0.61
Male	35 (46.1%)	90 (53.6%)	102 (55.1%)	28 (53.8%)	
Stage (TNM I–IV)					
I	9 (11.8%)	37 (22.2%)	37 (20.0%)	7 (13.5%)	0.47
II	25 (32.9%)	53 (31.7%)	52 (28.1%)	13 (25.0%)	
III	27 (35.5%)	56 (33.5%)	63 (34.1%)	23 (44.2%)	
IV	15 (19.7%)	21 (12.6%)	33 (17.8%)	9 (17.3%)	
pT					
1	0 (0)	10 (6.0%)	8 (4.4%)	2 (3.8%)	0.249
2	11 (15.1%)	35 (21.1%)	43 (23.6%)	8 (15.4%)	
3	52 (71.2%)	110 (66.3%)	112 (61.5%)	35 (67.3%)	
4	10 (13.7%)	11 (6.6%)	19 (10.4%)	7 (13.5%)	
pN					
0	37 (50.0%)	93 (56.4%)	95 (52.5%)	22 (42.3%)	0.064
1	23 (31.1%)	47 (28.3%)	53 (29.3%)	11 (21.2%)	
2	14 (18.9%)	26 (15.7%)	33 (18.2%)	19 (36.5%)	
pM					
0	61 (80.3%)	146 (87.4%)	152 (83.5%)	43 (82.7%)	0.50
1	15 (19.7%)	21 (12.6%)	30 (16.5%)	9 (17.3%)	
Grade					
Low	57 (79.2%)	147 (90.7%)	158 (89.8%)	43 (84.3%)	0.056
High	15 (20.8%)	15 (9.3%)	18 (10.2%)	8 (15.7%)	
Tumor location					
Right colon	46 (60.5%)	29 (17.3%)	54 (29.2%)	12 (23.1%)	< 0.001
Left colon	10 (13.2%)	53 (31.5%)	45 (24.3%)	7 (13.5%)	
Rectum	20 (26.3%)	86 (51.2%)	86 (46.5%)	33 (63.5%)	
Histology					
Non-mucinous	59 (84.3%)	144 (98.6%)	148 (92.5%)	44 (89.8%)	0.001
Mucinous	11 (15.7%)	2 (1.4%)	12 (7.5%)	5 (10.2%)	

*Using the Fisher’s exact test.

CMS-like, consensus molecular subtype resembling classification.

### Survival analysis

**Fig. 2:**
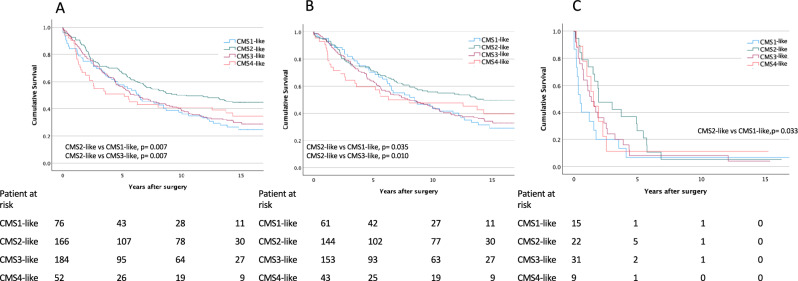
Overall survival of CRC patients according to CMS-resembling groups. A) CRC; B) local CRC; and C) metastasized CRC. Survival curves according to the Kaplan–Meier method, and compared using the log-rank test.

Patients with tumors resembling CMS2 had a better OS compared to those with tumors classified as CMS1-like (*p* = 0.007) and CMS3-like (*p* = 0.007; Fig. 2A). No differences in survival between patients with other CMS-resembling subtypes were found. Five-year OS for patients with CMS1-like tumors was 56.7% (95% confidence interval [CI] 45.6–67.8%), 67.0% (95% CI 59.6–74.2%) for CMS 2-like tumors, 56.4% (95% CI 48.9–63.8%) for CMS3-like tumors, and 51.0% (95% CI 37.3–64.2%) for CMS4-like tumors. In local CRC (stages I–III), patients with CMS2-like tumors also showed a better OS compared to those with CMS1-like (*p* = 0.035) and CMS3-like tumors (*p* = 0.010; Fig. 2B). In metastatic CRC, CMS2-like patients exhibited a better OS compared to CMS1-like patients (*p* = 0.033; Fig. 2C). No other differences were found (Fig. 2A–C). When assessed for DSS, we observed no differences between the CMS-resembling subtypes, either in local nor in metastatic disease (Supplementary Figs. 3A–C). However, the CMS1–4-like classifications used here are not direct replications of transcriptomic CMS subtypes, as they are based on surrogate markers. The distinction between CMS2-like and CMS3-like tumors, in particular, should be interpreted with caution due to the lack of transcriptomic validation.

### Subgroup analysis

Table 2 summarizes the univariate OS hazard ratios (HRs) for the CMS-resembling subtypes among the different clinicopathological groups. Based on the best prognosis found in the analysis above, the CMS2-resembling group was used as the reference value in the Cox regression analysis. Compared with CMS2-like in patients over 69 years, the CMS1-like (HR 2.05, 95% CI 1.33–3.16, *p* = 0.001) and CMS3-like (HR 1.57, 95% CI 1.57–2.26, *p* = 0.012) subgroups exhibited the worst survival (Supplementary Fig. 4A). In female patients, CMS1-resemblence (HR 1.8, 95% CI 1.13–2.86, *p* = 0.012) associated with a worse survival than CMS2-resemblance (Supplementary Fig. 4C). In male patients, the CMS4-like group (HR 1.84, 95% CI 1.10–3.08, *p* = 0.029) exhibited a worse prognosis compared with the CMS2-like group (Supplementary Fig. 4D). When comparing stages separately, CMS1-like exhibited a worse survival in stage I disease (HR 2.87, 95% CI 1.02–8.06, *p* = 0.039) and stage IV disease (HR 2.01, 95% CI 1.03–4.23, *p* = 0.033) compared with CMS2-like (Supplementary Figs. 4E and 4H). No differences were observed among locally advanced (T3–4) disease between groups, whereas CMS1-resemblance (HR 2.94, 95% CI 1.22–7.12, *p* = 0.011) and CMS3-resemblance (HR 2.31, 95% CI 1.19–4.48, *p* = 0.014) exhibited a worse prognosis compared with CMS2-resemblance among local disease (T1–2; Supplementary Fig. 4 M). In patients with low-grade tumors, CMS1-resemblance (HR 1.47, 95% CI 1.00–2.15, *p* = 0.047) and CMS3 (HR 1.46, 95% CI 1.09–1.95, *p* = 0.012) exhibited a worse prognosis compared with CMS2-resemblance (Supplementary Fig. 4 K). No differences in OS were observed between groups in right-sided colon or rectal tumors, but left-sided CMS1-resemblance patients (HR 2.95, 95% CI 1.32–6.60, *p* = 0.008) exhibited a worse prognosis compared with CMS2-resemblance (Supplementary Fig. 4 J).

**Table 2:** Subgroup univariate analysis of CMS-resembling groups. The CMS2-resembling subgroup served as the reference value.

Clinicopathological	CMS2-like	Number of	CMS1-like	Number of	CMS3-like	Number of	CMS4-like	Number of
Variable	HR (95% CI)	patients	HR (95% CI)	patients	HR (95% CI)	patients	HR (95% CI)	patients
Age								
< 69	1.00	92	1.24 (0.73–2.14)	38	1.13 (0.70–1.76)	74	1.41 (0.78–2.55)	28
≥ 69	1.00	74	2.05 (1.33–3.16)	38	1.57 (1.10–2.26)	110	1.58 (0.92–2.72)	24
Sex								
Female	1.00	77	1.80 (1.13–2.86)	41	1.47 (0.98–2.20)	83	1.05 (0.55–1.99)	24
Male	1.00	89	1.38 (0.85–2.23)	35	1.42 (0.98–2.06)	101	1.84 (1.10–3.08)	28
Stage (TNM I–IV)								
I	1.00	36	2.87 (1.02–8.06)	9	2.16 (0.96–4.84)	37	1.63 (0.44–6.02)	7
II	1.00	53	1.16 (0.63–2.15)	25	1.22 (0.74–2.02)	52	1.02 (0.42–2.48)	13
III	1.00	55	1.40 (0.80–2.44)	27	1.56 (1.00–2.42)	63	1.54 (0.85–2.80)	23
IV	1.00	21	2.01 (1.03–4.23)	15	1.19 (0.65–2.17)	32	1.36 (0.59–3.14)	9
pT								
1–2	1.00	44	2.94 (1.22–7.12)	11	2.31 (1.19–4.48)	51	1.61 (0.57–4.51)	10
3–4	1.00	120	1.25 (0.86–1.80)	62	1.36 (1.00–1.84)	130	1.44 (0.93–2.22)	42
pN								
N0	1.00	92	1.67 (1.01–2.76)	37	1.39 (0.92–2.10)	95	1.33 (0.68–2.59)	22
N+	1.00	72	1.41 (0.89–2.23)	37	1.59 (1.10–2.30)	85	1.43 (0.86–2.36)	30
pM								
M0	1.00	144	1.49 (1.02–2.19)	61	1.49 (1.09–2.02)	152	1.42 (0.90–2.25)	43
M+	1.00	21	2.07 (1.02–4.19)	15	1.49 (0.80–2.76)	29	1.35 (0.58–3.12)	9
Grade (WHO)								
Low	1.00	145	1.47 (1.00–2.15)	57	1.46 (1.09–1.95)	157	1.42 (0.92–2.20)	43
High	1.00	15	1.14 (0.49–2.65)	15	1.73 (0.75–3.97)	18	1.14 (0.39–3.36)	8
Tumor location								
Right colon	1.00	29	1.52 (0.84–2.76)	46	1.48 (0.83–2.67)	54	1.52 (0.63–3.71)	12
Left colon	1.00	43	2.95 (1.32–6.60)	10	1.70 (0.98–2.93)	44	1.50 (0.52–4.31)	7
Rectum	1.00	84	1.19 (0.66–2.15)	20	1.28 (0.87–1.87)	86	1.32 (0.80–2.19)	33

CMS-like, consensus molecular subtype resembling classification; CI, confidence interval; HR, hazard ratio; TNM, tumor, node, metastasis; WHO, World Health Organization.

### Multivariable analysis

In the multivariable analysis, an older age (HR 2.54, 95% CI 1.98–2.37), stage III (HR 2.42, 95% CI 1.64–3.56), and stage IV disease (HR 5.63, 95% CI 3.65–8.68) served as independent indicators of a poorer prognosis. CMS1-resemblance represented an independent predictor of a poor prognosis compared with CMS2-resemblance (HR 1.49, 95% CI 1.02–2.17; Table 3).

**Table 3:** Uni- and multivariable cox regression analysis for overall survival in colorectal cancer.

Clinicopathological variable	HR (95% CI)	*p* value	HR (95% CI)	*p* value
Age	Univariable		Multivariable	
< 69	1.00		1.00	
≥ 69	2.47 (1.98–3.10)	< 0.001	2.51 (1.97–3.19)	< 0.001
Sex				
Female	1.00			
Male	1.11 (0.89–1.37)	0.36		
Stage (TNM IV)				
I	1.00		1.00	
II	1.30 (0.91–1.86)	0.145	1.58 (1.06–2.34)	0.024
III	2.03 (1.45–2.84)	< 0.001	2.52 (1.73–3.69)	< 0.001
IV	5.84 (4.01–8.49)	< 0.001	6.51 (4.29–9.88)	< 0.001
Grade (WHO)				
Low	1.00			
High	1.17 (0.85–1.61)	0.33		
Tumor location				
Right colon	1.00			
Left colon	0.83 (0.61–1.13)	0.23		
Rectum	0.97 (0.76–1.26)	0.85		
CMS-like group				
CMS2-like	1.00		1.00	
CMS1-like	1.57 (1.13–2.20)	0.008	1.56 (1.12–2.18)	0.009
CMS3-like	1.45 (1.10–1.90)	0.008	1.25 (0.93–1.61)	0.15
CMS4-like	1.45 (0.98–2.17)	0.067	1.42 (0.95–2.12)	0.087

CMS-like, consensus molecular subtype resembling classification; CI, confidence interval; HR, hazard ratio; TNM, tumor, node, metastasis; WHO, World Health Organization.

## Discussion

In this study, the CMS2-resembling subtype associated with the best overall prognosis both in local and metastatic CRC when patients were divided into the four CMS-resembling groups based on different clinical characteristics using an artificial intelligence–assisted method.

The distribution of CMS-resembling groups was roughly similar to previous IHC-based reports where the patient cohorts comprised CRC patients at all stages of disease. One primary difference consisted of a lower proportion of CMS4-resembling tumors (13%) in our series compared with 43%^15^ and 24%^16^ in previously published reports. The staging distribution between cohorts was similar, although our cohort consisted of a significantly higher proportion of rectal tumors (48.7%) compared with 31.5%^15^ and 20.3%^16^ in other reports. Compared with the transcriptomic classification^18^, the proportion of CMS3-resembling tumors in our cohort was higher (38.7% vs 14.9%), while the proportion of CMS4-resembling tumors was lower (11.7% vs 26.4%).

In agreement with previous reports, CMS1-resembling tumors were more common in the right hemicolon and CMS2-resembling tumors were more common in the left hemicolon and rectum^16,18^. Among CMS1- and CMS4-resembling tumors, a mucinous histology appeared more common, an observation consistent with findings reported by Li et al.^16^. CMS4-resembling tumors were reportedly more common in advanced disease^18^. However, we found no clear association between CMS-resembling group and stage of disease.

In the subgroup analysis, the better prognosis for CMS2-resembling tumors compared with other groups was more common among older patients and also among less advanced and aggressive tumors—that is, low-grade tumors. A similar effect of CMS2-resembling was also observed among left-sided colon tumors. By contrast, in more aggressive and advanced disease the effect of the CMS-resembling class on prognosis was less clear. A similar effect was reported by Trinh et al.^15^, where differences in OS were more conclusive in cohorts including either all stages or stage II patients instead of stage IV patients alone. The subgroups were relatively small in our study and, thus, this result must be interpreted with caution. However, a similar trend was observed in all previously mentioned situations involving the T-stage and tumor differentiation. Our IHC-based classification may aid in patient stratification for therapy, particularly in identifying CMS2-resembling patients who may have a more favorable prognosis. However, further studies are needed to establish whether CMS2-resembling tumors might be candidates for de-escalated treatment strategies.

In the OS analysis, patients with CMS2-resembling tumors exhibited the best prognosis in local disease, which appears to agree with previous reports^10^. Yet, we observed no conclusively better prognosis for CMS1-resemblance or worse prognosis for CMS4-resemblance in local disease, a phenomenon previously reported^10^. In metastatic disease, CMS1-resemblance associated with a poorer prognosis compared with CMS2-resemblance, a finding consistent with previous studies^10^. Because we observed no significant differences in DSS, it seems that at least part of the differences found in OS result from the selection of younger patients in the CMS2-resembling subgroup, even though the CMS2-resembling subgroup exhibited a better OS in the multivariable analysis as well.

Beyond the established TNM classification, additional prognostic factors such as tumor budding and the ImmunoScore^19^ are gaining recognition in CRC stratification. Tumor budding, defined as the presence of single tumor cells or small clusters at the invasive front, has been associated with poor prognosis and increased metastatic potential^20^. The ImmunoScore, which quantifies immune infiltration in the tumor microenvironment, has been linked to favorable outcomes, particularly in MSI-high tumors^19^. Future studies should explore whether CMS2-resembling tumors, which exhibit a more differentiated and epithelial-like phenotype, correlate with lower tumor budding and a higher immune score. This would provide additional insights into the biological behavior of CMS-resembling subtypes and their potential prognostic implications.

CMS1-resembling tumors are characterized by MSI, but MSI alone does not distinguish between sporadic cases and those associated with Lynch syndrome (LS). To refine CMS1 classification, additional molecular markers, such as BRAF V600E mutation status, should be considered. LS cases typically lack this mutation^21^. This distinction is clinically relevant, as LS-associated CRCs often present at a younger age and may have different therapeutic implications, including heightened sensitivity to immune checkpoint inhibitors^22^. Future work should aim to integrate germline testing and somatic mutation profiling to further delineate CMS1-resembling tumors into biologically and clinically distinct subsets.

Sex-based differences in MSI tumors have been reported in gastric cancer, where females with MSI tumors exhibit improved survival compared to males^23^. Although our analysis did not reveal a significant sex-dependent effect within CMS1-resembling tumors , this remains an area of interest, as hormonal and immune-related factors may influence CRC progression differently in men and women. Further large-scale studies incorporating sex as a stratification factor may help clarify whether CMS1-resembling tumors exhibit gender-based prognostic differences similar to those observed in gastric cancer.

The strengths of this study include the large patient cohort with detailed clinicopathological parameters available and the long-term follow-up period. The limitations to this study consist of the retrospective setting and the lack of gene expression data on our patients. Our approach builds upon previous IHC classifiers^15,16^ and extends their utility with CNN-based scoring. However, the original classifier by Trinh et al. was not explicitly designed to classify CMS groups but rather de Sousa et al.’s subtypes^8^ This may introduce misclassification, particularly in CMS1-resembling subtype, where MSI alone is an imperfect classifier. Moreover, the lack of a transcriptomic reference for differentiating CMS2-CMS3-resembling subtypes remains a limitation. Convoluted neural networks represent a practical tool for analyzing larger patient cohorts and provide an easy-to-repeat analysis of IHC stainings. Because different IHC panels^13^ have been proposed for clinical use, a study comparing different classification methods, their overlap, and prognostic and predictive capabilities in a large CRC cohort is warranted in future research.

To conclude, we demonstrated that all four CMS-resembling groups can be identified by immunohistochemistry using convoluted neural networks. In our cohort, patients with CMS2–resembling tumors exhibited the best prognosis.

## Supplementary Information

Supplementary Information 1.

Supplementary Information 2.

Supplementary Information 3.

Supplementary Information 4.

Supplementary Information 5.

## Data Availability

The datasets generated and analysed during the current study are available from the corresponding author on reasonable request.
