# Supplementary material for: An immunohistochemistry-based classification of colorectal cancer resembling the consensus molecular subtypes using convolutional neural networks
Source: Sci Rep. 2025 May 31;15:19105. doi: 10.1038/s41598-025-03618-z (PMC12125322; doi:10.1038/s41598-025-03618-z)

Supplementary Figure 1

Workflow of developing the semi-quantitative classification system using convoluted neural networks. 1) Preparation and staining of tissue samples; 2) Digitalization into high resolution images; 3) Uploading into the Aiforia® platform; 4) Annotation on a representative (5%) subset of the whole dataset and repetative trainning of the convoluted neural networks (CNN); 6) Validation of each CNN with external validators; 7) Extraction of data to decide CMS-resembling-status of each sample for further statistical analysis

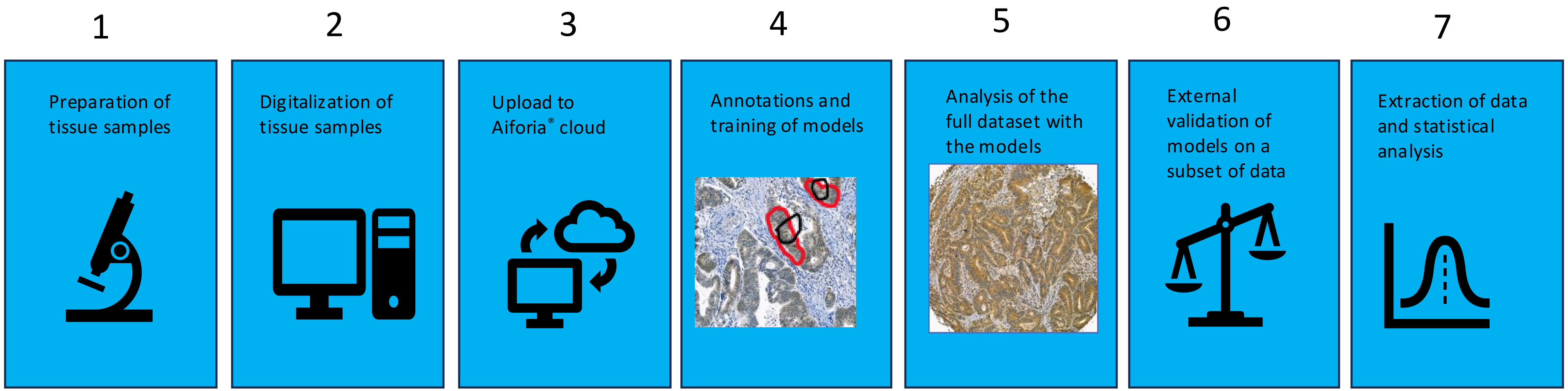

Supplement: Supplementary file 1 — Supplementary Information 1. [file 41598_2025_3618_MOESM1_ESM.pdf]
