# Supplementary material for: An immunohistochemistry-based classification of colorectal cancer resembling the consensus molecular subtypes using convolutional neural networks
Source: Sci Rep. 2025 May 31;15:19105. doi: 10.1038/s41598-025-03618-z (PMC12125322; doi:10.1038/s41598-025-03618-z)
Supplement: Supplementary file 2 — Supplementary Information 2. [file 41598_2025_3618_MOESM2_ESM.pdf]

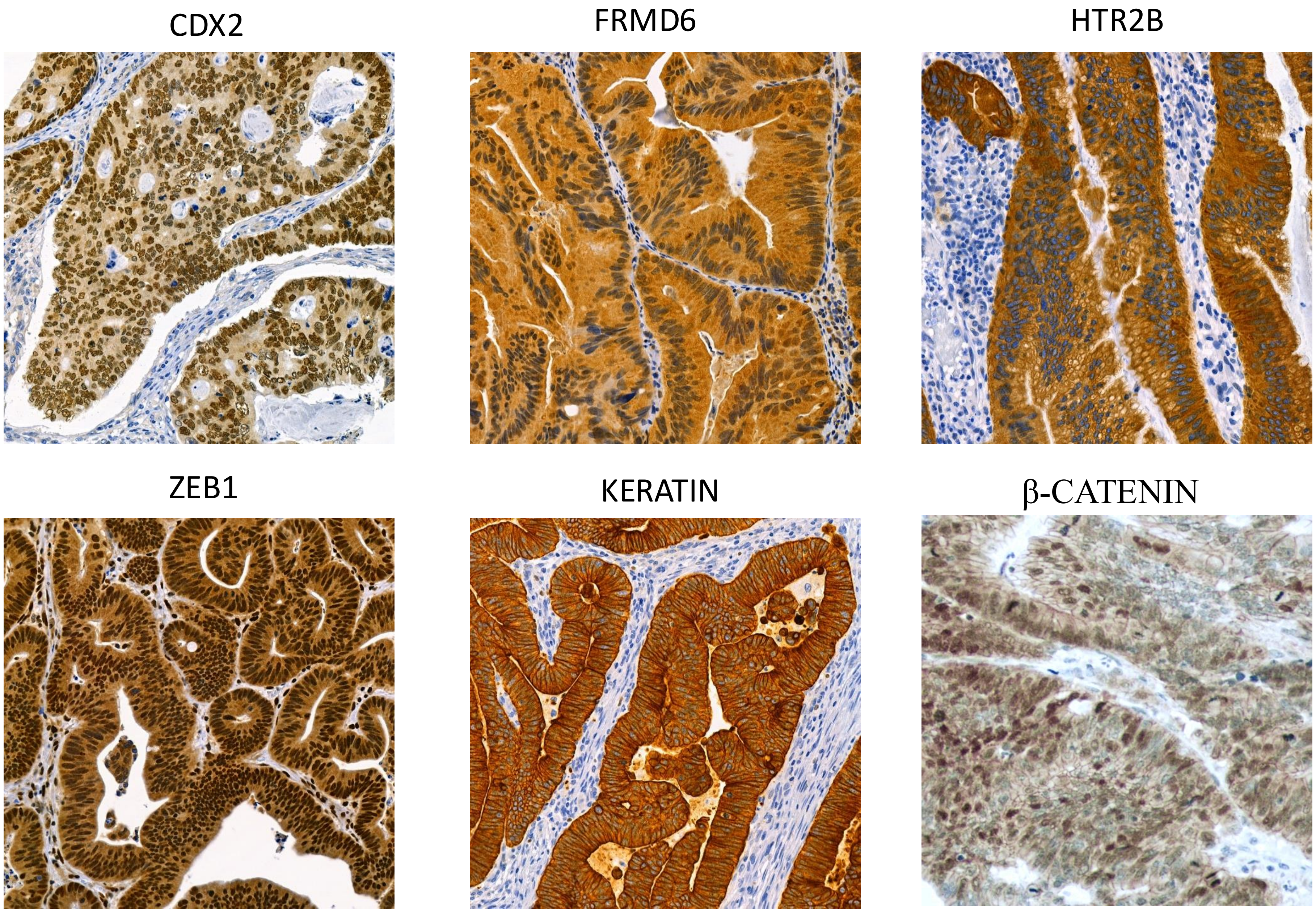

Supplementary Figure 2 Representative images of the CDX, FRMD6, HTR2B, ZEB1, Cytokeratin, and β-catenin immunohistochemistry. Original magnification: 20x
