# Supplementary material for: An immunohistochemistry-based classification of colorectal cancer resembling the consensus molecular subtypes using convolutional neural networks
Source: Sci Rep. 2025 May 31;15:19105. doi: 10.1038/s41598-025-03618-z (PMC12125322; doi:10.1038/s41598-025-03618-z)
Supplement: Supplementary file 3 — Supplementary Information 3. [file 41598_2025_3618_MOESM3_ESM.pdf]

Supplementary Figure 3. Disease specific survival of CRC according to CMS-resembling groups A) CRC; B)stage I-III CRC; C) stabe IV CRC. Survival curves according to the Kaplan–Meier method, and compared with the log-rank test.

A B C

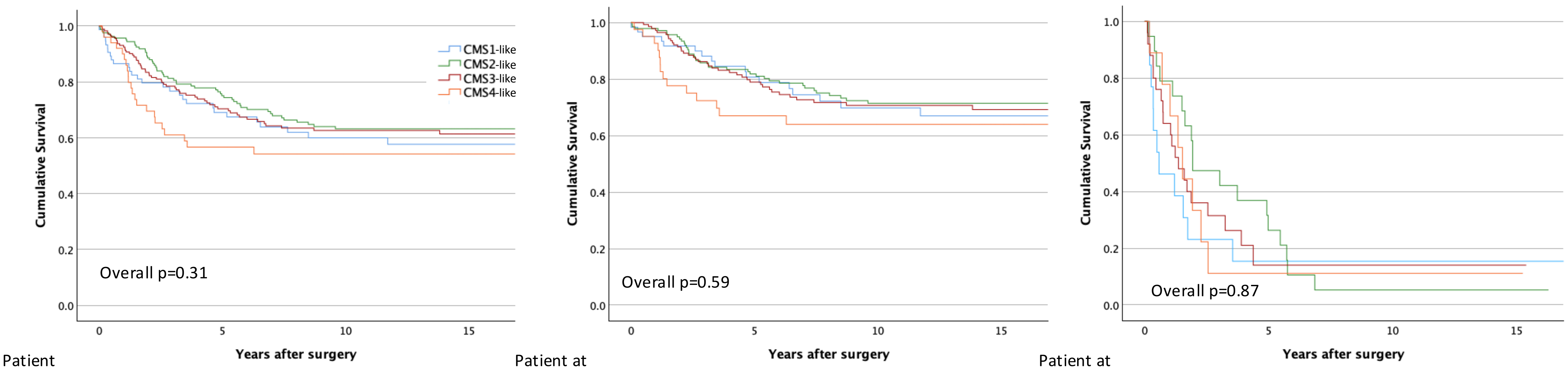

A)

Patient at risk				
CMS1-like	76	43	28	11
CMS2-like	166	107	78	30
CMS3-like	184	95	64	27
CMS4-like	52	26	19	9

B)

Patient at risk				
CMS1-like	61	42	27	11
CMS2-like	144	102	77	30
CMS3-like	153	93	63	27
CMS4-like	43	25	19	9

C)

Patient at risk				
CMS1-like	15	1	1	0
CMS2-like	22	5	1	0
CMS3-like	31	2	1	0
CMS4-like	9	1	0	0
