# Supplementary material for: An immunohistochemistry-based classification of colorectal cancer resembling the consensus molecular subtypes using convolutional neural networks
Source: Sci Rep. 2025 May 31;15:19105. doi: 10.1038/s41598-025-03618-z (PMC12125322; doi:10.1038/s41598-025-03618-z)
Supplement: Supplementary file 4 — Supplementary Information 4. [file 41598_2025_3618_MOESM4_ESM.pdf]

Supplementary figure 4

Overall survival of CRC patients according to CMS-resembling groups A) under 69 years; B) 69 years or older; C) females; D) males; E) TNM stage I; F) stage II; G) stage III; H) stage IV; I) right colon cancer; J) left colon cancer; K) rectal cancer; L) low grade; M)high grade; N) T1-2 tumor; O) T3-4 tumor Survival curves according to the Kaplan–Meier method, and compared with the log-rank test.

A

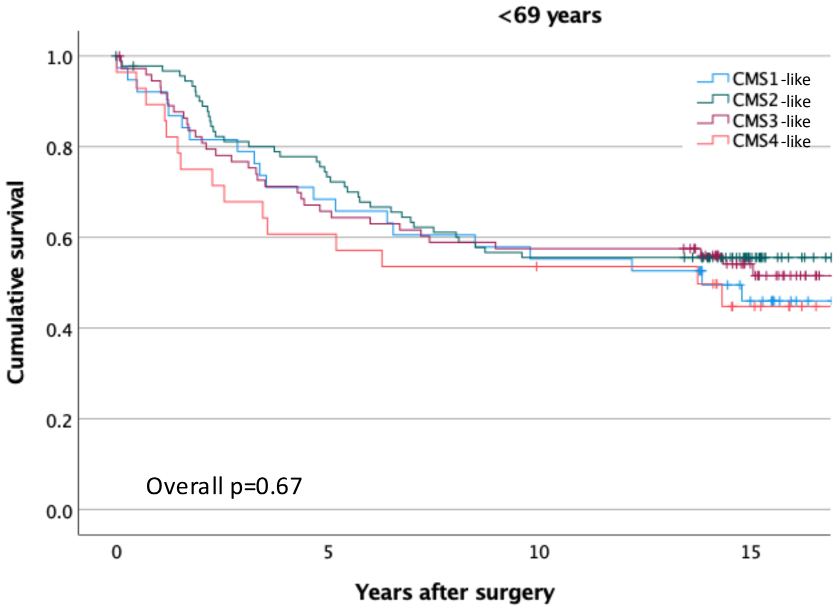

B

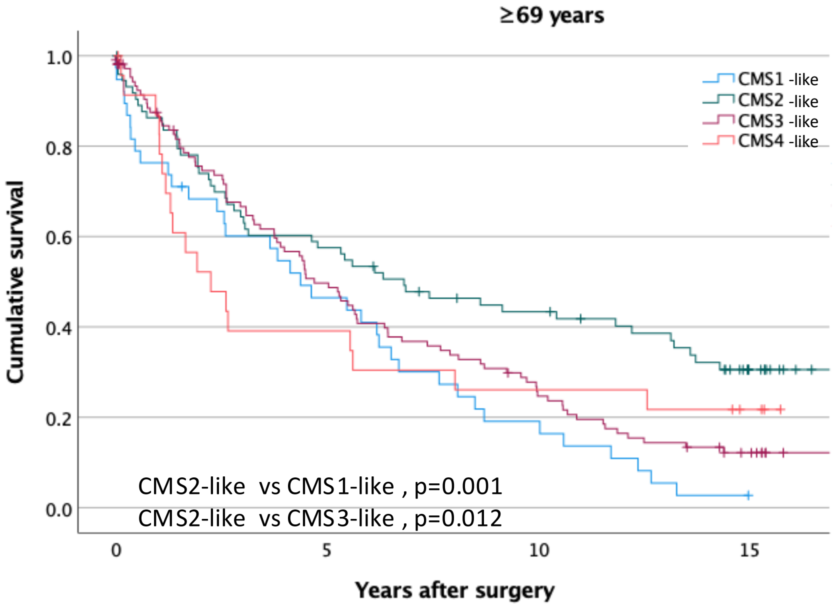

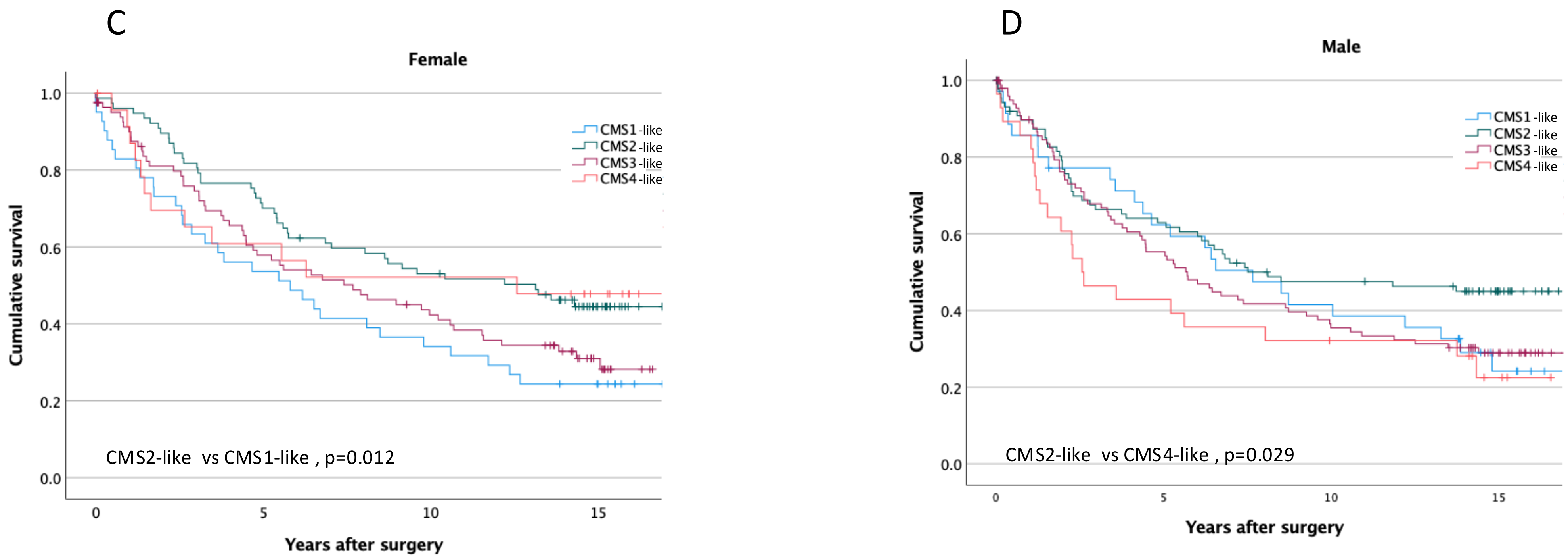
C
Female
Cumulative survival
1.0
0.8
0.6
0.4
0.2
0.0
CMS1-like
CMS2-like
CMS3-like
CMS4-like
CMS2-like vs CMS1-like , p=0.012
0
5
10
15
Years after surgery
D
Male
Cumulative survival
1.0
0.8
0.6
0.4
0.2
0.0
CMS1-like
CMS2-like
CMS3-like
CMS4-like
CMS2-like vs CMS4-like , p=0.029
0
5
10
15
Years after surgery

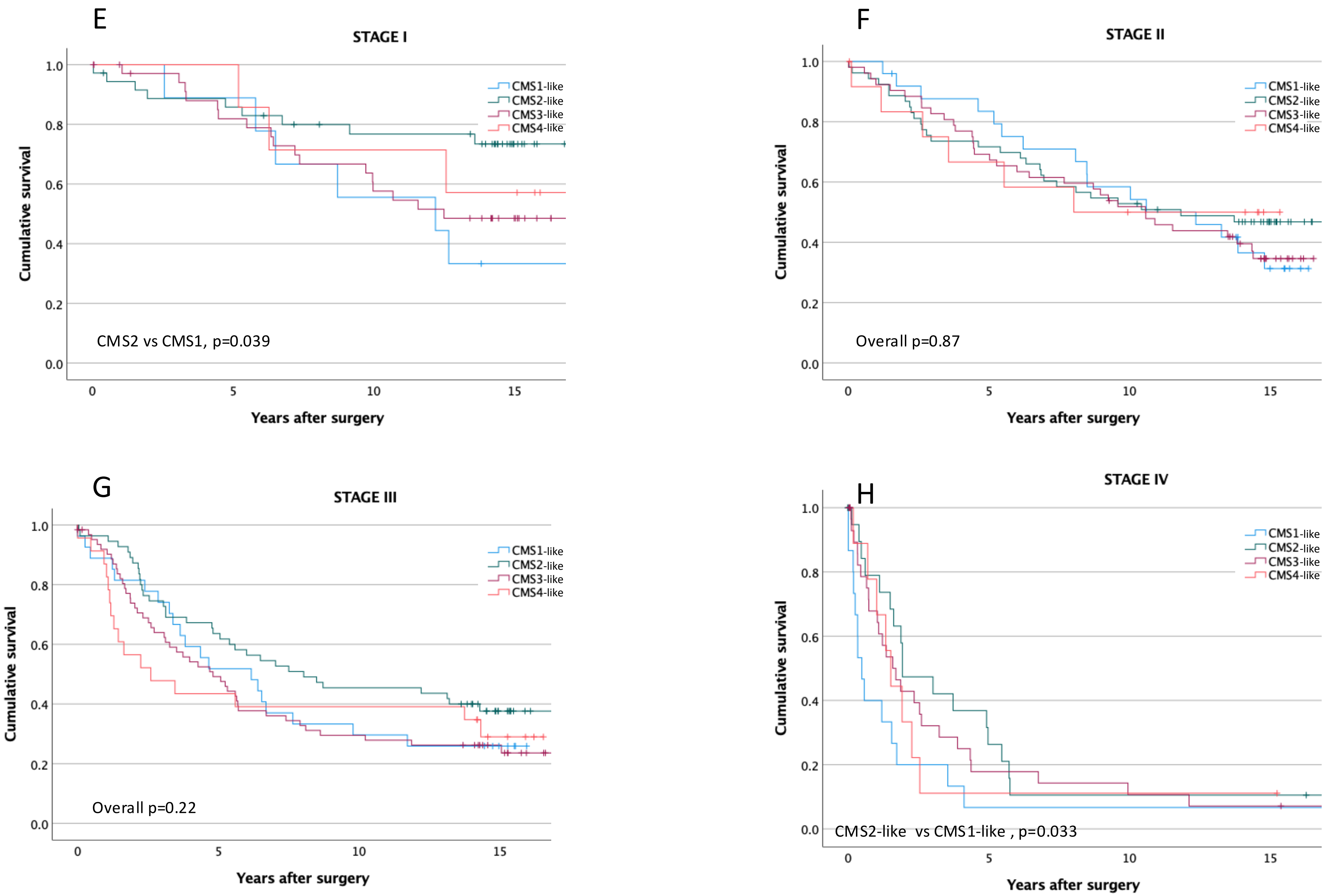
E
STAGE I
CMS1-like
CMS2-like
CMS3-like
CMS4-like
Cumulative survival
Years after surgery
CMS2 vs CMS1, p=0.039
F
STAGE II
CMS1-like
CMS2-like
CMS3-like
CMS4-like
Cumulative survival
Years after surgery
Overall p=0.87
G
STAGE III
CMS1-like
CMS2-like
CMS3-like
CMS4-like
Cumulative survival
Years after surgery
Overall p=0.22
H
STAGE IV
CMS1-like
CMS2-like
CMS3-like
CMS4-like
Cumulative survival
Years after surgery
CMS2-like vs CMS1-like , p=0.033

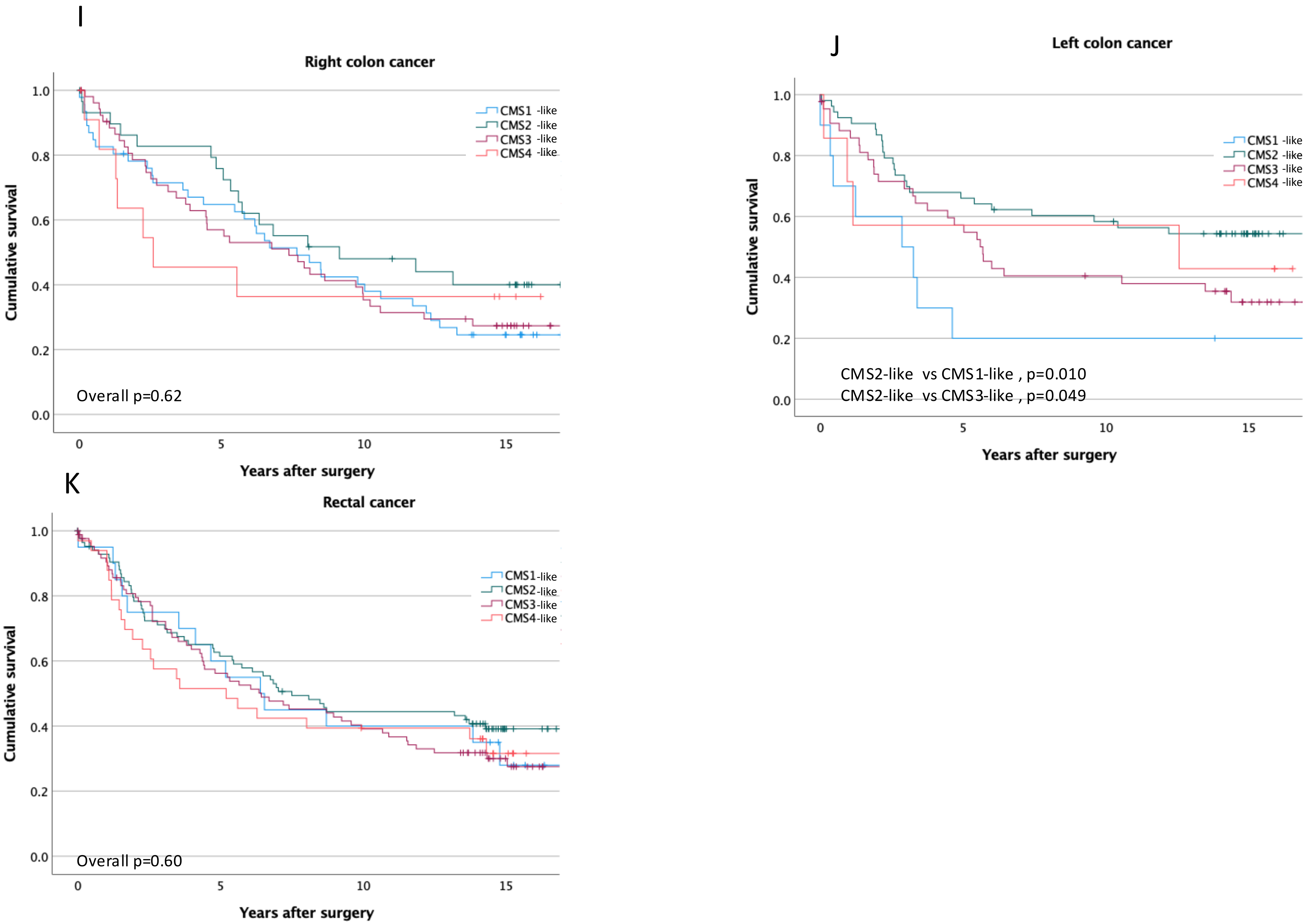
I
Right colon cancer
CMS1 -like
CMS2 -like
CMS3 -like
CMS4 -like
Overall p=0.62
Cumulative survival
Years after surgery
J
Left colon cancer
CMS1 -like
CMS2 -like
CMS3 -like
CMS4 -like
CMS2-like vs CMS1-like , p=0.010
CMS2-like vs CMS3-like , p=0.049
Cumulative survival
Years after surgery
K
Rectal cancer
CMS1-like
CMS2-like
CMS3-like
CMS4-like
Overall p=0.60
Cumulative survival
Years after surgery

L

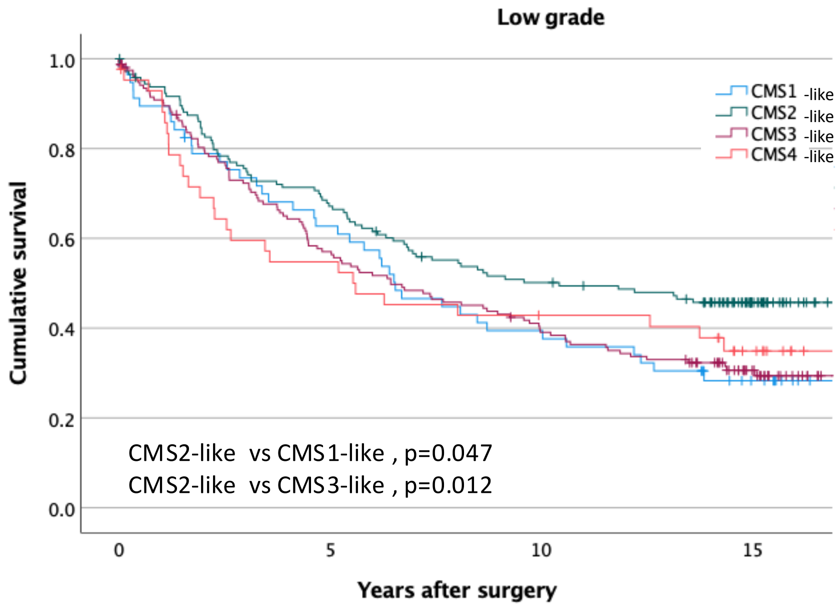
Low grade
Cumulative survival
1.0
0.8
0.6
0.4
0.2
0.0
CMS1 -like
CMS2 -like
CMS3 -like
CMS4 -like
CMS2-like vs CMS1-like , p=0.047
CMS2-like vs CMS3-like , p=0.012
0
5
10
15
Years after surgery

M

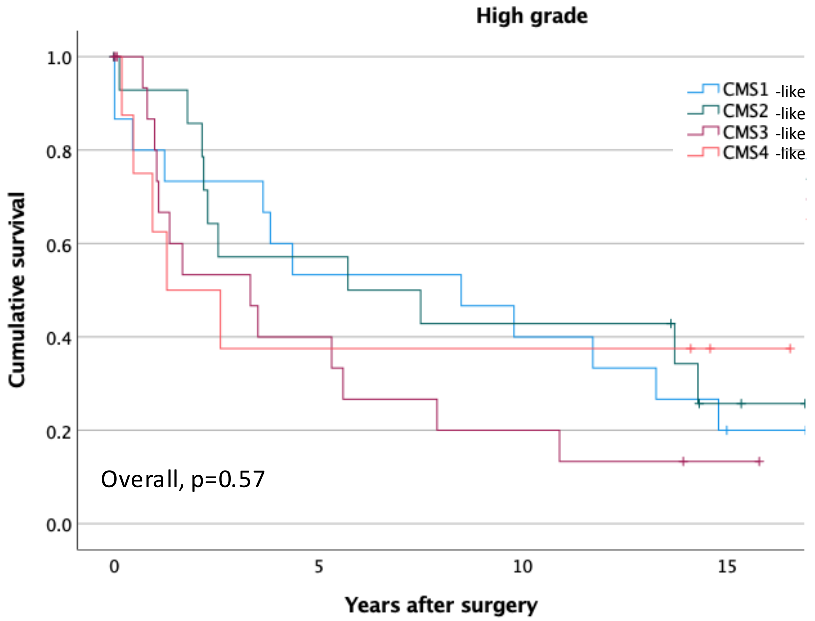
High grade
Cumulative survival
1.0
0.8
0.6
0.4
0.2
0.0
CMS1 -like
CMS2 -like
CMS3 -like
CMS4 -like
Overall, p=0.57
0
5
10
15
Years after surgery

N

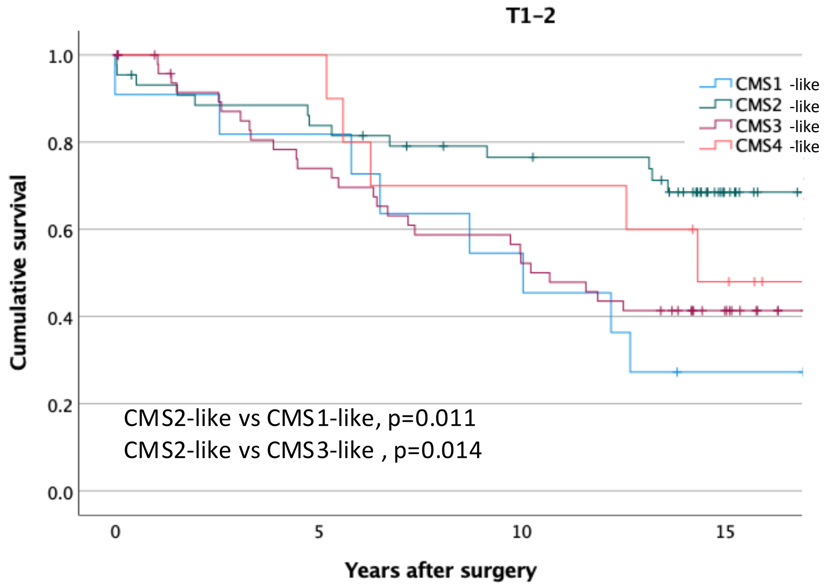
T1–2
1.0
0.8
0.6
0.4
0.2
0.0
Cumulative survival
0
5
10
15
Years after surgery
CMS1 -like
CMS2 -like
CMS3 -like
CMS4 -like
CMS2-like vs CMS1-like, p=0.011
CMS2-like vs CMS3-like , p=0.014

O

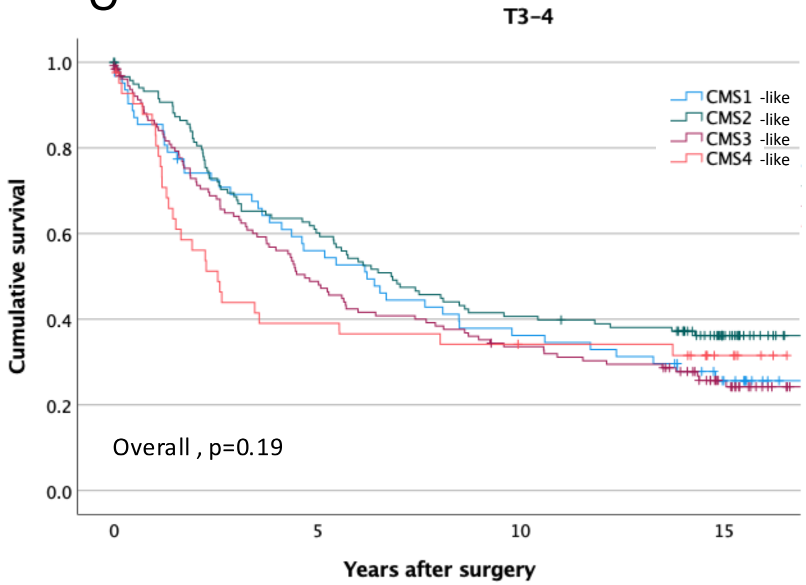
T3–4
1.0
0.8
0.6
0.4
0.2
0.0
Cumulative survival
0
5
10
15
Years after surgery
CMS1 -like
CMS2 -like
CMS3 -like
CMS4 -like
Overall , p=0.19
