# Supplementary material for: An immunohistochemistry-based classification of colorectal cancer resembling the consensus molecular subtypes using convolutional neural networks
Source: Sci Rep. 2025 May 31;15:19105. doi: 10.1038/s41598-025-03618-z (PMC12125322; doi:10.1038/s41598-025-03618-z)
Supplement: Supplementary file 5 — Supplementary Information 5. [file 41598_2025_3618_MOESM5_ESM.docx]

**Supplementary table 1** Antibodies used to determine CMS-resembling groups

|  | **Antibody** | **Incubation-time** |
| --- | --- | --- |
| **CDX2** | Novus NB100-2136, 1:500 | 60min |
| **FMRD6** | Ab 218209 (Ab) 1:300 | Overnight |
| **ZEB1** | Sigma HPA027524, 1:300 60mio | 60min |
| **KER** | Thermo Scientific, clone AE1/AE3, diluted 1:200 | overnight |
| **HTR2B** | Sigma HPA012867 1:500 | overnight |
| **β-catenin** | Invitrogen 180226, 373mg/ml, 1:300 | 60min |

**Supplementary table 2** Characteristics of AI‐models

| **Model** | **CDX2** | **FRMD6** | **KER** | **HTR2B** | **ZEB1** |
| --- | --- | --- | --- | --- | --- |
| **Stain** | IHC for CDX2 | IHC for FRMD6 | IHC for KER | IHC for HTR2B | IHC for ZEB1 |
| **Training annotations per layer** | Layer 1: 70  Layer 2: 130 | Layer 1: 130  Layer 2: 170 | Layer 1: 80  Layer 2: 140 | Layer 1: 80  Layer 2: 110 | Layer 1: 90  Layer 2: 105  Layer 3: 60 |
| **Iterations in model** | 2010 | 2232 | 2316 | 2212 | 2200 |
| **Layer 1** | Area  Tissue | Area  Tissue | Area  Tissue | Area  Tissue | Area  Tissue |
| **Layer 2** | Area  Epithelial intensity and percentage of epithelial immunoreactivity | Area  Epithelial intensity of immunoreactivity | Area  Epithelial intensity and percentage of epithelial immunoreactivity | Area  Epithelial intensity and percentage of epithelial immunoreactivity | Area  Epithelium |
| **Layer 3** | - | - | - | - | Area  Nuclear area and immunoreactivity |

**Supplementary table 3** Validation between CNNs and human validators.

|  | **CNN to Validator 1** | **CNN to Validator 2** | **CNN to Validator 3** | **Between validators** |
| --- | --- | --- | --- | --- |
| **Keratin** |  |  |  |  |
| F1-score (%) tissue | 99.00 | 98.64 | 98.65 | 98.83 |
| F1-score% epithelium | 99.03 | 99.12 | 99.14 | 99.26 |
| Matching intensity (%) | 53.4 | 61.5 | 69.2 | 48.7 |
| **HRT2B** |  |  |  |  |
| F1-score % tissue | 99.13 | 99.37 | 99.10 | 99.31 |
| F1-score % epithelium | 98.55 | 98.02 | 98.12 | 98.52 |
| Matching intensity (%) | 69.2 | 53.8 | 69.2 | 61.5 |
| **CDX2** |  |  |  |  |
| F1-score % tissue | 99.06 | 99.13 | 98.00 | 99.56 |
| F1-score % epithelium | 98.90 | 98.85 | 98.44 | 98.84 |
| Matching intensity (%) | 81.2 | 72.7 | 72.7 | 65.2 |
| **FRMD6** |  |  |  |  |
| F1-score % tissue | 98.88 | 98.61 | 98.74 | 98.84 |
| Matching intensity (%) | 76.9 | 61.5 | 61.5 | 50.0 |
| **ZEB1** |  |  |  |  |
| F1-score % tissue | 98.13 | 98.59 | 98.12 | 99.09 |
| F1score % epithelium | 97.43 | 97.43 | 97.70 | 97.84 |
| F1-score % nuclei | 84.41 | 91.03 | 83.23 | 83.30 |
